# Association between dietary inflammation index and albuminuria: results from the National Health and Nutrition Examination Survey

**DOI:** 10.3389/fnut.2024.1361890

**Published:** 2024-04-15

**Authors:** Ling Ding, Haipeng Guo, Chao Zhang, Bo Jiang, Shuo Zhang, Jian Zhang

**Affiliations:** Department of Laboratory Medicine, The First Hospital of Qiqihar, Qiqihar, Heilongjiang, China

**Keywords:** dietary inflammation index, albuminuria, National Health and Nutrition Examination Survey (NHANES), cross-sectional study, population-based study

## Abstract

**Background:**

The Dietary Inflammation Index (DII) is a tool for evaluating the potential for dietary inflammation, and inflammation is a major cause of exacerbation in chronic kidney disease. Our study aimed to investigate the relationship between DII and albuminuria.

**Methods:**

Data were obtained from the 2005–2018 National Health and Nutrition Examination Survey (NHANES) after excluding pregnant, minors, and missing data of urinary albumin-creatinine ratio (ACR), estimated glomerular filtration rate (eGFR), and DII were enrolled in our study. Albuminuria was defined as ACR > 30 mg/g. DII was calculated and divided into tertiles. After fully adjusted, multivariate logistic regression analysis and subgroup analysis were performed to investigate the association between DII and albuminuria.

**Results:**

A total of 22,607 participants including 2,803 (12.40%) with and 19,804 (87.60%) without albuminuria were enrolled in our study. The albuminuria increased with the increasing DII tertiles (Tertile 1: 10.81%; Tertile 2: 12.41%; Tertile 3:13.97%, *P* < 0.001). After fully adjusting for covariates, multivariate logistic regression showed that the higher the DII, the greater the odds of albuminuria (OR = 1.19; 95% CI, 1.00–1.41, *P* < 0.001). Subgroup analysis and interaction test of participants found that the positive correlation between DII and albuminuria was not significantly related to gender, age, BMI, hypertension, diabetes, and eGFR (*P* for interaction >0.05).

**Conclusion:**

Elevated DII is associated with high odds of albuminuria. Further large-scale prospective studies are still needed to analyze the role of DII in albuminuria.

## Introduction

Increased urinary albumin excretion is the most important clinical manifestation of early nephropathy (1). The urinary albumin creatinine ratio (ACR) is a reliable indicator to assess glomerular injury and albuminuria. ACR can be used in the diagnosis of early chronic kidney disease (CKD), diabetic nephropathy, and hypertensive nephropathy (2). Albuminuria was defined as ACR>30 mg/g. Albuminuria is not only an independent predictor of CKD progression but also a target of treatment in CKD therapy (3–5). Studies have found that moderately increased albuminuria (ACR = 30–300mg/g) occurs in 5%-19% of the general population (6). Matsushita et al. (7) found that albuminuria was significantly associated with all-cause mortality and cardiovascular mortality in the general population. Therefore, the public health interventions can be implemented to attenuate CKD by studying the risk factors of albuminuria.

The Dietary Inflammation Index (DII) is used as a standardized scoring tool to assess the impact of diet on inflammation. This index was first calculated by Cavicchia et al. and further updated by Shivappa et al. (8, 9). DII correlates with a variety of inflammatory factors including C-reactive protein (CRP), tumor necrosis factor (TNF)-α, interleukin (IL)-6, IL−1β, IL−4, and IL−10, etc. Therefore, a higher DII represents a pro-inflammatory diet while a negative DII represents an anti-inflammatory diet. DII has been demonstrated to be associated with CKD, rheumatoid arthritis, kidney stones, diabetes, and hyperuricemia (10–14). DII is also associated with lipid metabolism, thyroid function, and sex hormone secretion (15–17). Dyslipidemia due to decreased lipoprotein catabolism in patients with nephrotic syndrome. Parathyroid hormone is elevated in patients with renal insufficiency due to decreased renal excretion of calcium. The reticular band of the adrenal cortex secretes adrenal sex hormones. Thus, DII may affect renal function by regulating lipid metabolism as well as the endocrine system. DII is an independent risk factor for all-cause and cardiovascular mortality (18).

Numerous studies have shown that inflammation leads to a decline in kidney function. CRP is a risk factor for acute kidney injury (AKI) and CKD by leading to the decline of the estimated glomerular filtration rate (eGFR) (19). TNF-α directly affects kidney function by regulating kidney hemodynamics and excretory functions (20). Serum IL-1β levels were significantly higher in patients with albuminuria than without-albuminuria in patients with diabetes, and the concentration and ACR showed a significant negative correlation (21). However, the association between DII and albuminuria has not been clearly articulated. Therefore, our purpose was to explore whether elevated DII is associated with an increased odds of albuminuria by investigating the participants in the National Health and Nutrition Examination Survey (NHANES).

## Materials and methods

### Survey description

All cross-sectional data for this study were obtained from NHANES, an ongoing nationally representative sample recruited every 2 years by the National Center for Health Statistics (NCHS) through multistratified, multistage probability sampling across the United States to monitor the nutritional status and health risk factors of the civilian population in the non-institutionalized United States (22). Demographic data, dietary data, and health condition data were obtained through in-home interviews; physical and laboratory examinations were obtained through mobile examination centers (MECs) with participants. We retrieved data of demographics data, dietary data, examination data, laboratory data, and questionnaire data. The protocol for the National Health and Nutrition Examination Survey study was approved by the National Center for Health Statistics Research Ethics Review Board (NHANES 2005–2006, NHANES 2007–2008, NHANES 2009–2010: Protocol #2005–06; NHANES 2011–2012, NHANES 2013–2014, NHANES 2015–2016: Protocol #2011–17; NHANES 2017–2018): Protocol #2011–17 (Effective through October 26, 2017); Protocol #2018–01 (Effective beginning October 26, 2017). The valid informed consent was obtained from all participants for NHANES. All the protocols, detailed experimental design, and data are available at: https://www.cdc.gov/nchs/nhanes/. The findings and conclusions in this paper are those of the authors and do not necessarily represent the views of the Research Data Center, the National Center for Health Statistics, or the Centers for Disease Control and Prevention. Data collection for National Center for Health Statistics (NCHS) was approved by the NCHS Research Ethics Review Board. Analysis of de-identified data from the survey is exempt from the federal regulations for the protection of human research participants. Analysis of restricted data through the NCHS Research Data Center is also approved by the NCHS ERB.

**Figure 1:**
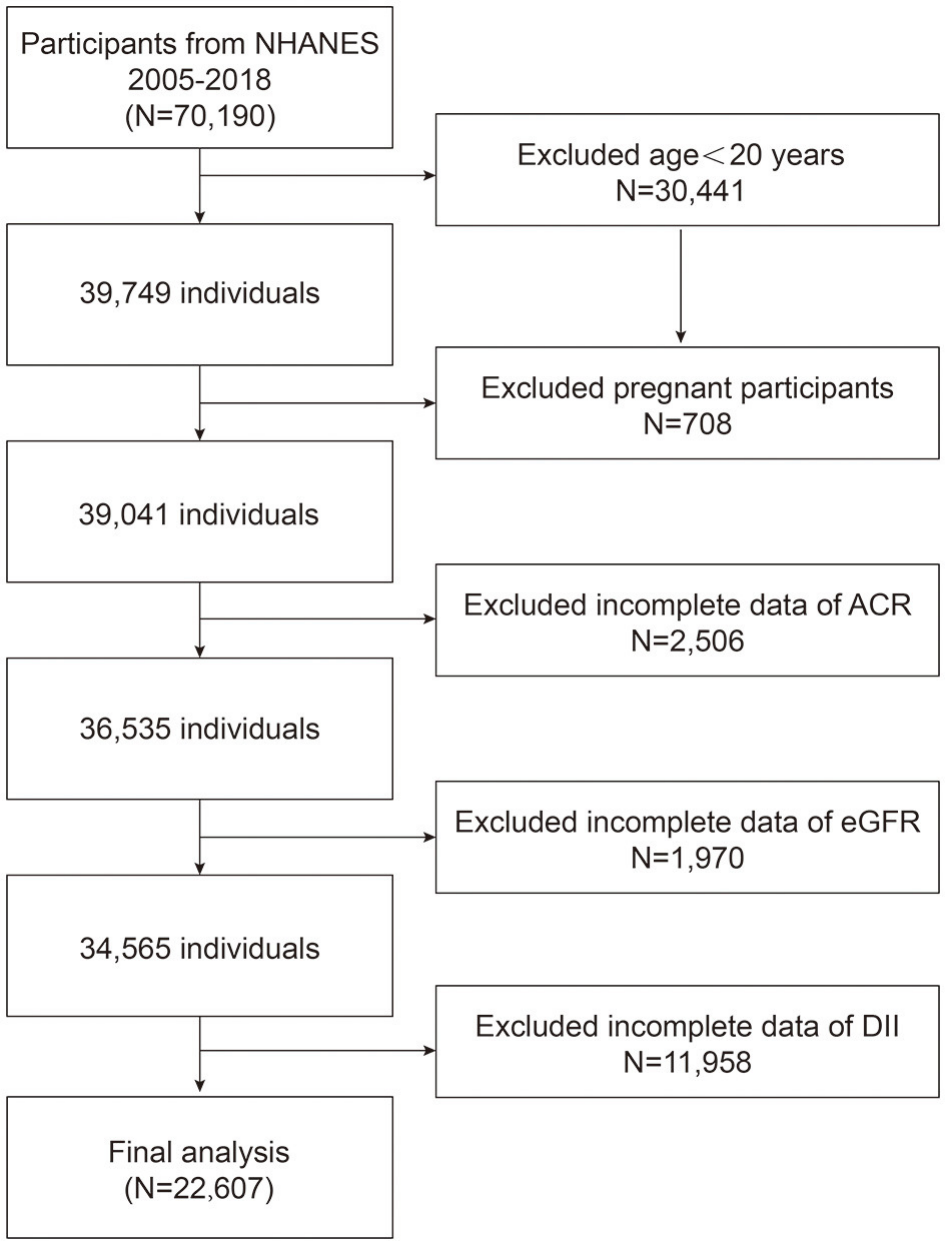
Flowchart of the sample from NHANES 2005–2018.

We recruited 7 NHANES cycles from 2005 to 2018 to assess the association between DII and albuminuria. A total of 70,190 participants were enrolled in the study at first. We included 22,607 eligible participants in the final study based on exclusion criteria: age < 20 years (*n* = 30,441); pregnancy was defined by self-report pregnant now (*n* = 708); missing data ACR (*n* = 2,506), eGFR was calculated from the data about gender, age, and SCr (*n* = 1,970), and DII was calculated from 24 h dietary history interview (*n* = 11,958) (Figure 1).

Sampling weights are usually used to ensure the data enrolled are representative and unbiased when the data from complex investigations. However, adjusting the variables used to calculate sampling weights in regression analyses may lead to excessive adjustment bias and reduce the accuracy of the analysis. Therefore, we determined to demonstrate our results without the sampling weights.

### Definition of dietary inflammation index

The DII score was developed by Shivappa et al. (9) to assess the potential level of inflammation from dietary components through dietary intake recorded by participants' 24-h dietary history interviews (data from USDA's Food and Nutrient Database for Dietary Studies). In our study, DII was calculated from the intake of 28 dietary components and nutrients, including protein, carbohydrates, cholesterol, total fat, saturated fat, monounsaturated fatty acids (MUFAs), n-3 fatty acids, polyunsaturated fatty acids (PUFAs), n-6 fatty acids, vitamin A/B12/C/D/E, thiamine (vitamin B1), riboflavin (vitamin B2), folic acid, zinc, selenium, beta-carotene, alcohol, fiber, Fe, Mg, caffeine, niacin, and energy (23). First, the average value of dietary components and nutrients consumed by each participant during the day (midnight to midnight) was subtracted from the global average per capita daily intake of dietary components and nutrients. The resulting value was divided by the standard deviation of the global average per capita daily dietary component or nutrient intake and multiplied by the associated inflammatory effect index. The above calculation results in a Z-score, which is used to express the “standardized global average.” Individual DII scores were obtained by multiplying the total inflammation score for each dietary component or nutrient after converting the Z-scores into percentiles (to reduce the effect of right skew). An elevated DII score indicates an increased pro-inflammatory effect of the diet, while a negative score indicates an anti-inflammatory effect of the diet.

### Definition of albuminuria

Data for urinary albumin and urinary creatinine were obtained from the laboratory data of the 2005–2018 NHANES participants tested at MECs. Urinary albumin was measured by a solid-phase fluorescence immunoassay; urinary creatinine was measured by a modified Jaffe kinetic method. All methodological information was obtained from the Laboratory Procedure Manual of NHANES. ACR was calculated by dividing the urine albumin concentration by the urine creatinine concentration. The diagnosis of albuminuria is defined as an ACR >30 mg/g (24). In our study, albuminuria was defined as an outcome variable.

### Covariates

We also included covariates that may have influenced the association between DII and albuminuria in participants in the study, including age (years); gender (male/female); race/ethnicity (Mexican America/Other Hispanic/Non-Hispanic White /Non-Hispanic Black/Other); education (less than high school/high school or equivalent/college or above/not recorded); systolic blood pressure (SBP; mmHg); diastolic blood pressure (DBP; mmHg); body mass index was calculated as weight (kilograms) divided by height (m) squared and divided (BMI; kg/m^2^); waist circumference (cm); hypertension was defined as having been diagnosed by a physician or taking hypertension medication (yes/no); diabetes were defined as self-reported doctor diagnosis of diabetes, HbA1c ≥6.5%, FPG ≥7.0 mmol/L, postprandial 2-h plasma glucose≥11.1 mmol/L from an oral glucose tolerance test or use of insulin or oral hypoglycemic medication (yes/no); smoking status (current/former/never/not recorded); serum albumin (ALB; g/L); serum Blood Urea Nitrogen (BUN; mmol/L); serum Uric Acid (UA; μmol/L); alanine transaminase (ALT; U/L); aspartate transaminase (AST; U/L); total cholesterol (mmol/L); high-density cholesterol (HDL-C; mmol/L); and eGFR (ml/min/1.73 m^2^). The estimated glomerular filtration rate (eGFR; ml/min/1.73 m^2^) was calculated based on the formula: eGFR = 142^*^min (standardized Scr/K, 1)^α^^*^max (standardized Scr/K, 1)^−1.200^^*^0.9938^Age^^*^1.012 [if female] (K = 0.7 (females) or 0.9 (males); α = −0.241 (females) or −0.302 (males)) according to the CKD Epidemiology Collaboration (CKD-EPI) creatinine equation (25). Serum creatinine (SCr; mg/dl) was determined by the Jaffe rate method and calibrated by standardized isotope dilution mass spectrometry (6).

### Statistical analysis

Statistical analysis was performed using R version 3.4.3 (http://www.R-project.org, The R Foundation) and Empower software (www.empowerstats.com; X&Y Solutions, Inc., Boston, MA). The enrolled participants were stratified into three groups based on DII tertiles (T1-T3) in the sensitivity analysis. Analysis of variation in baseline characteristics across participants grouped by DII tertiles was performed using linear regression models for continuous variables and the chi-square test for the categorical variables. Continuous variables in the study are presented as mean ± standardized values. Categorical variables are presented as frequencies and percentages. *P*-values for continuous variables, obtained by Kruskal Wallis test, and if the count variable theoretical number < 10, *P*-values obtained by Fisher's exact probability test. The *P* < 0.05 was determined statistically significant. The baseline characteristics across participants to test the relationship between critical variables and albuminuria to target special populations.

To investigate the relationship between DII and albuminuria. We used multifactorial logistic regression to test for an association between DII and albuminuria in three different models. Model 1 was unadjusted for covariates, Model 2 was adjusted for age, gender, race/ethnicity, and Model 3 was further adjusted for education, SBP, DBP, BMI, waist circumference, hypertension, diabetes, smoking status, ALB, BUN, UA, ALT, AST, total cholesterol, HDL-C, and eGFR. We conducted the monofactor analysis to perform the association between each covariate and albuminuria in the fully adjusted models. The OR of albuminuria was each unit increase in continuous variables and compared with the reference group for categorical variables. The smoothed fitted curves to assess the linear relationship between DII and albuminuria in Model 3.eGFR was categorized as < 60, 60–89.9, and ≥90 ml/min/1.73m^2^, and BMI was categorized as < 25 (normal weight), 25–29.9 (overweight), and ≥30 kg/m^2^ (obese) in subgroup analysis. By stratification factors: sex (male/female); age (< 60/≥60 years); BMI (< 25/25–29.9/≥30 kg/m^2^); hypertension (yes/no); diabetes (yes/no); eGFR (< 60/60–89.9/≥90 ml/min/1.73 m^2^) were subjected to subgroup analysis and interaction tests with stratification factors as potential effect modifiers to test the heterogeneity of associations between subgroups.

## Results

### Baseline characteristics of participants by DII tertile

Based on inclusion criteria, a total of 22,607 participants from NHANES 2005 to 2018 were enrolled, of whom with an average age of 50.26 ± 17.68 years. The 49.78% participants were male and 50.22% were female. A total 12.40% of the participants were categorized as albuminuria overall. The percentage of albuminuria increased with the DII tertiles (Tertile 1: 10.81%; Tertile 2: 12.41%; Tertile 3: 13.97%; *P* < 0.001). There were statistically significant differences in age, gender, race, education, SBP, DBP, BMI, waist circumference, hypertension, diabetes, smoking status, serum Albumin, serum Blood Urea Nitrogen, serum Uric Acid, eGFR, ALT, AST, ACR among the DII tertiles (-5.2811–0.8623, 0.8628–2.63771, 2.6378–5.79464). All baseline characteristics were summarized in Table 1.

**Table 1:** Baseline characteristics of study population according to dietary inflammatory index tertiles.

	**Tertiles of dietary inflammatory index**				***P*-value**
	**Total**	**Tertile 1**	**Tertile 2**	**Tertile 3**	
*N*	22,607	7,536	7,535	7,536	
Age (years)	50.26 ± 17.68	50.01 ± 17.13	50.05 ± 17.65	50.71 ± 18.23	0.028
Gender					< 0.001
Male (%)	11,253 (49.78%)	4,547 (60.34%)	3,818 (50.67%)	2,888 (38.32%)	
Female (%)	11,354 (50.22%)	2,989 (39.66%)	3,717 (49.33%)	4,648 (61.68%)	
Race/ethnicity					< 0.001
Mexican America (%)	3,962 (17.53%)	1,410 (18.71%)	1,375 (18.25%)	1,177 (15.62%)	
Other Hispanic (%)	2,184 (9.66%)	681 (9.04%)	749 (9.94%)	754 (10.01%)	
Non-Hispanic White (%)	9,997 (44.22%)	3,500 (46.44%)	3,288 (43.64%)	3,209 (42.58%)	
Non-Hispanic Black (%)	4,497 (19.89%)	1,137 (15.09%)	1,508 (20.01%)	1,852 (24.58%)	
Other (%)	1967 (8.70%)	808 (10.72%)	615 (8.16%)	544 (7.22%)	
Education					< 0.001
Less than high school (%)	5,759 (25.47%)	1,571 (20.85%)	1,894 (25.14%)	2,294 (30.44%)	
High school or equivalent (%)	5,333 (23.59%)	1,531 (20.32%)	1,786 (23.70%)	2,016 (26.75%)	
Collage or above (%)	11,500 (50.87%)	4,429 (58.77%)	3,853 (51.13%)	3,218 (42.70%)	
Not recorded (%)	15 (0.07%)	5 (0.07%)	2 (0.03%)	8 (0.11%)	
SBP (mmHg)	125.51 ± 19.11	124.53 ± 17.99	125.74 ± 19.03	126.26 ± 20.22	< 0.001
DBP (mmHg)	70.53 ± 13.21	71.02 ± 12.71	70.60 ± 13.18	69.95 ± 13.70	< 0.001
BMI (kg/m^2^)	29.28 ± 6.82	28.60 ± 6.32	29.41 ± 6.90	29.83 ± 7.17	< 0.001
Waist circumference (cm)	99.65 ± 16.21	98.49 ± 15.56	99.99 ± 16.37	100.49 ± 16.63	< 0.001
Hypertension (%)	8,110 (35.87%)	2,478 (32.88%)	2,703 (35.87%)	2,929 (38.87%)	< 0.001
Diabetes (%)	2,943 (13.02%)	826 (10.96%)	1,030 (13.67%)	1,087 (14.42%)	< 0.001
Smoking status					< 0.001
Current (%)	3,819 (16.89%)	949 (12.59%)	1,236 (16.40%)	1,634 (21.68%)	
Former (%)	872 (3.86%)	313 (4.15%)	272 (3.61%)	287 (3.81%)	
Never (%)	5,665 (25.06%)	2,060 (27.34%)	1,907 (25.31%)	1,698 (22.53%)	
Not recorded (%)	12,251 (54.19%)	4,214 (55.92%)	4,120 (54.68%)	3,917 (51.98%)	
Serum Albumin (g/L)	42.14 ± 3.37	42.62 ± 3.34	42.18 ± 3.28	41.62 ± 3.41	< 0.001
Serum Blood Urea Nitrogen (mmol/L)	4.99 ± 2.13	5.09 ± 1.93	4.99 ± 2.08	4.87 ± 2.35	< 0.001
Serum Uric Acid (μmol/L)	326.06 ± 86.04	327.21 ± 82.83	326.06 ± 85.27	324.90 ± 89.87	0.003
eGFR (ml/min/1.73m^2^)	93.53 ± 22.41	94.89 ± 20.73	93.65 ± 22.33	92.07 ± 23.98	< 0.001
ALT (U/L)	25.23 ± 19.28	26.64 ± 20.11	25.50 ± 19.68	23.56 ± 17.84	< 0.001
AST (U/L)	25.45 ± 16.57	26.13 ± 13.98	25.55 ± 19.06	24.69 ± 16.25	< 0.001
Total cholesterol (mmol/L)	5.01 ± 1.07	5.01 ± 1.07	5.01 ± 1.07	5.01 ± 1.08	0.952
HDL-C (mmol/L)	1.37 ± 0.42	1.38 ± 0.42	1.37 ± 0.42	1.36 ± 0.42	0.108
ACR (mg/g)	45.79 ± 345.36	39.06 ± 323.78	46.58 ± 373.38	51.74 ± 336.93	< 0.001
Albuminuria (%)	2,803 (12.40%)	815 (10.81%)	935 (12.41%)	1,053 (13.97%)	< 0.001

Data are presented as mean ± SD or n (%).

### Association of albuminuria with DII

Our results indicated that higher DII was significantly associated with an elevated probability of albuminuria in the crude model (OR = 1.08; 95% CI, 1.05–1.10, *P* < 0.001), minimally adjusted model (OR = 1.07; 95% CI, 1.05–1.09, *P* < 0.001), and fully adjusted model (OR = 1.05; 95% CI, 1.02–1.10, *P* < 0.01). In fully adjusted model, each unit increase in DII was associated with 5% increased odds of albuminuria. We further converted DII from a continuous variable to a categorical variable by tertiles. After full adjustment, the risk of albuminuria in participants was 19% higher and statistically significant in the highest DII tertile compared to the lowest DII tertile (OR = 1.19; 95% CI, 1.00–1.41, *P* < 0.05). The participants in the medium DII tertile also demonstrated higher odds of albuminuria compared to the lowest DII tertile; however, this correlation was not statistically significant (OR = 1.12; 95% CI, 0.95–1.33, *P* > 0.05) (Table 2).

**Table 2:** Association between dietary inflammatory index and albuminuria.

	**OR**^**a**^ **(95%CI**^**b**^**)**, ***p*****-value**		
	**Crude model (Model 1)** ^c^	**Minimally adjusted model(Model 2)** ^d^	**Fully adjusted model (Model 3)** ^e^
**Continuous**			
	1.08 (1.05, 1.10) < 0.0001	1.07 (1.05, 1.09) < 0.0001	1.05 (1.02, 1.10) 0.0060
**Categories**			
Tertile 1	Reference	Reference	Reference
Tertile 2	1.17 (1.06, 1.29) 0.0023	1.16 (1.04, 1.28)0.0057	1.12 (0.95, 1.33) 0.1626
Tertile 3	1.34 (1.21, 1.48) < 0.0001	1.28 (1.16, 1.42) < 0.0001	1.19 (1.00, 1.41) 0.0437
*P* for trend	< 0.0001	< 0.0001	0.0424

In sensitivity analysis, DII was converted from a continuous variable to a categorical variable (tertiles). ^a^OR: odds ratio. ^b^95% CI: 95% confidence interval. ^c^Model 1: no covariates were adjusted. ^d^Model 2: adjusted for gender, age, race. ^e^Model 3: adjusted for gender, age, race, education, systolic blood pressure, diastolic blood pressure, body mass index, waist circumference, hypertension, diabetes, smoking status, Serum Albumin, Serum Blood Urea Nitrogen, Serum Uric Acid, eGFR, ALT, AST, Total cholesterol, and HDL-C.

In the fully adjusted model, age, race, education, SBP, DBP, BMI, waist circumference, hypertension, diabetes, smoking status, Serum Albumin, Serum Blood Urea Nitrogen, Serum Uric Acid, eGFR, AST, HDL-C associated with the increased odds of albuminuria significantly. The odds of albuminuria increased by 4% for each year of age (*P* < 0.001); non-Hispanic whites were 12% less likely to have albuminuria compared to Mexican Americans (*P* < 0.05); the odds of albuminuria decreased by 29% for those with a high school or equivalent compared to those with less than a high school education (*P* < 0.001), and decreased by 43% for those with a college degree or above (*P* < 0.001); the odds of albuminuria for the non-hypertension and non-diabetes compared to hypertension and diabetes were 68% (*P* < 0.001) and 79% (*P* < 0.001); the odds of albuminuria increased by 2% for each unit increase in BMI and waist circumference (*P* < 0.001), and increased by 3% for each unit increase in SBP (*P* < 0.001), decreased by 1% for each unit increase in DBP (*P* < 0.001). Increased serum albumin, eGFR, and HDL-C reduced the risk of albuminuria, and increased serum blood urea nitrogen, serum uric acid, and AST escalated the odds of albuminuria (Table 3).

**Table 3:** Multivariate logistic regression models of albuminuria.

**Variables**	**OR^a^ (95%CI^b^)**	***p*-value**
Age (years)	1.04 (1.04, 1.04)	< 0.0001
Female ( vs. male)	0.99 (0.91, 1.07)	0.7236
Race/ethnicity ( vs. Mexican America)		
Other Hispanic	0.85 (0.73, 1.00)	0.0539
Non-Hispanic White	0.88 (0.79, 0.99)	0.0272
Non-Hispanic Black	1.07 (0.95, 1.22)	0.2623
Other	0.85 (0.72, 1.00)	0.0541
Education ( vs. Less than high school)		
High school or equivalent	0.71 (0.64, 0.79)	< 0.0001
Collage or above	0.57 (0.52, 0.63)	< 0.0001
Not recorded	0.36 (0.05, 2.71)	0.3192
SBP (mmHg)	1.03 (1.03, 1.04)	< 0.0001
DBP (mmHg)	0.90 (0.89, 0.91)	< 0.0001
BMI (kg/m^2^)	1.02 (1.02, 1.03)	< 0.0001
Waist circumference (cm)	1.02 (1.01, 1.02)	< 0.0001
Hypertension (no vs. yes)	0.32 (0.30, 0.35)	< 0.0001
Diabetes (no vs. yes)	0.21 (0.19, 0.23)	< 0.0001
Smoking status ( vs. current)		
Former	0.75 (0.58, 0.96)	0.0208
Never	1.31 (1.16, 1.48)	< 0.0001
Serum Albumin (g/L)	0.90 (0.89, 0.91)	< 0.0001
Serum Blood Urea Nitrogen (mmol/L)	1.25 (1.23, 1.27)	< 0.0001
Serum Uric Acid (μmol/L)	1.00 (1.00, 1.00)	< 0.0001
eGFR (ml/min/1.73 m^2^)	0.97 (0.97, 0.98)	< 0.0001
ALT (U/L)	1.00 (1.00, 1.00)	0.4968
AST (U/L)	1.00 (1.00, 1.01)	0.0004
Total cholesterol (mmol/L)	0.98 (0.95, 1.02)	0.3113
HDL-C (mmol/L)	0.84 (0.76, 0.92)	0.0004

^a^OR: odds ratio. ^b^95% CI: 95% confidence interval. The unit for continuous variables and the reference group for categorical variables are provided next to the variables. The OR of albuminuria was each unit increase in continuous variables and compared with the reference group for categorical variables.

**Figure 2:**
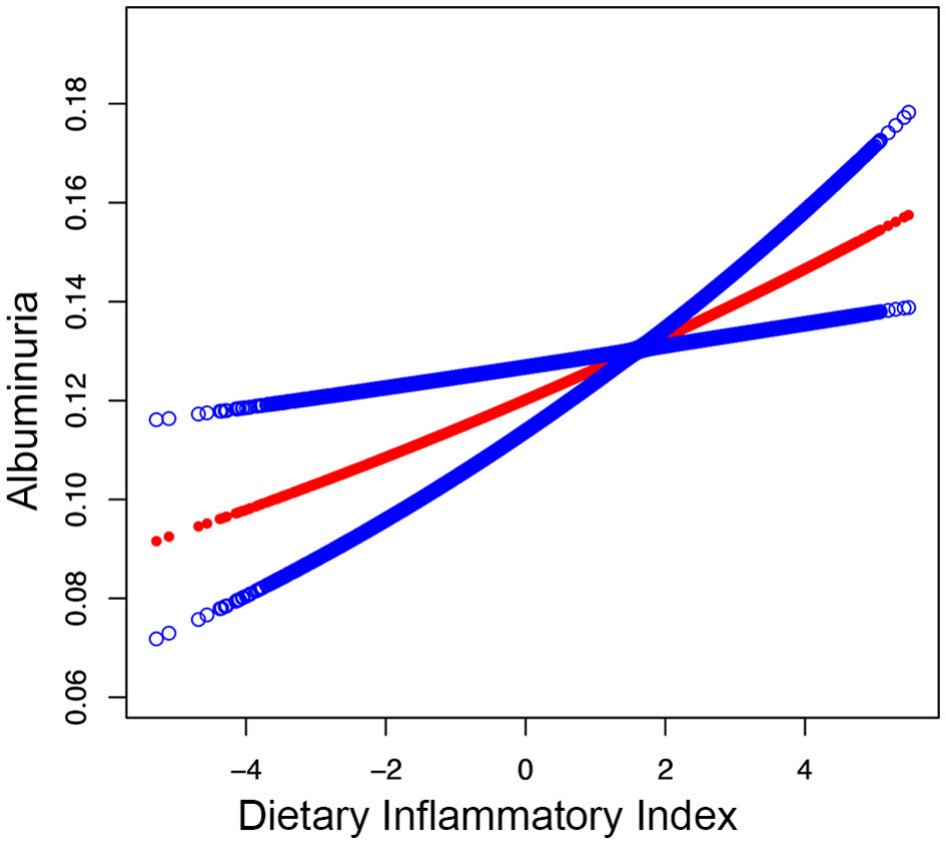
Smooth curve fitting for DII and albuminuria. A linear relationship between DII and albuminuria was detected by the generalized model.

Moreover, the smooth curve fitting showed no non-linear relationship between DII and albuminuria in participants (Figure 2).

### Subgroup analysis

**Figure 3:**
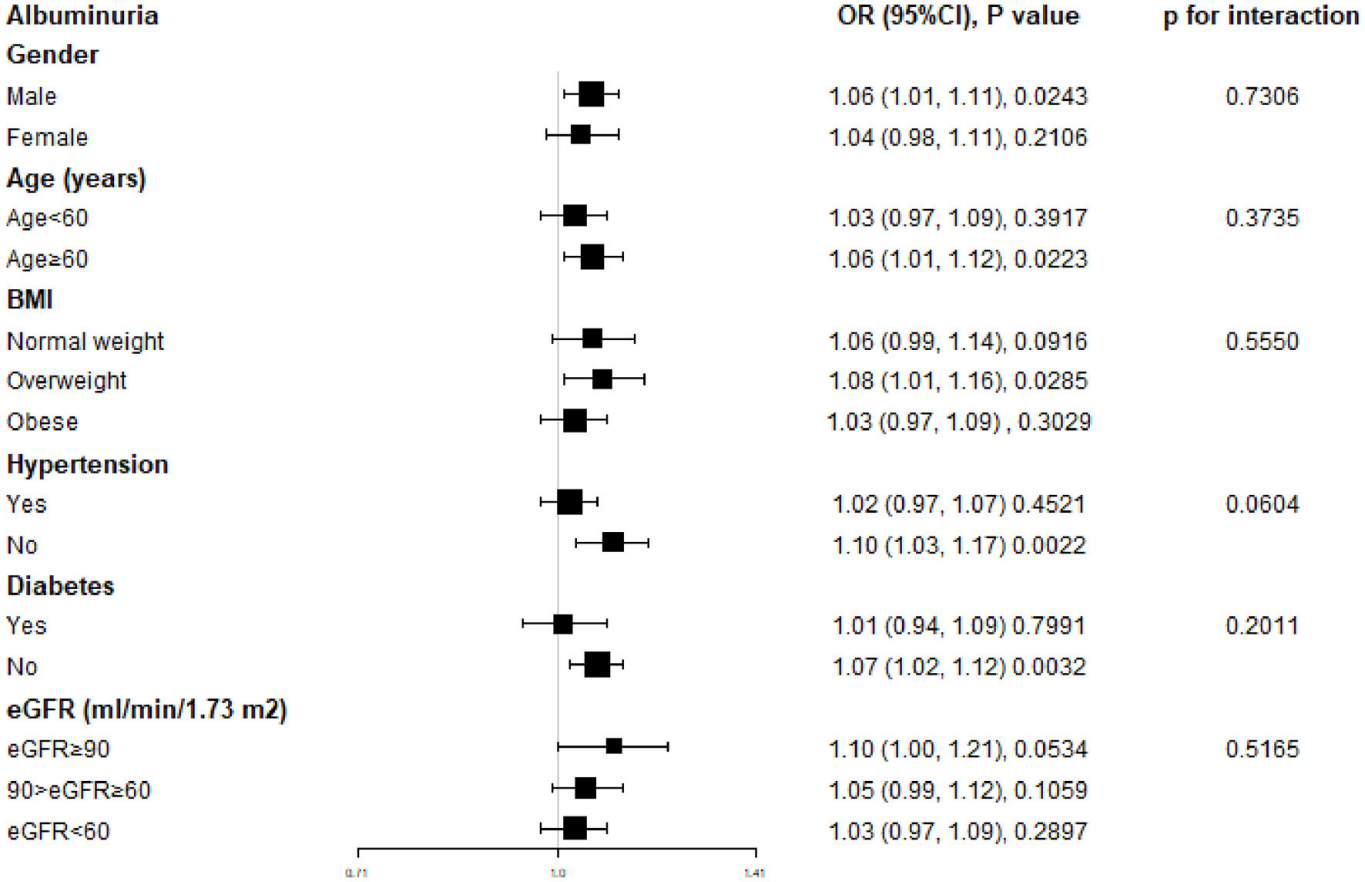
Subgroup analysis for the association between DII and albuminuria.

To assess whether the relationship between DII and albuminuria is stable across diverse demographics, we performed a subgroup analysis. The results showed that DII was positively associated with albuminuria across subgroups and was more significant in participants who were male, age ≥ 60, overweight, non-hypertension, and non-diabetes (*P* < 0.01), however, there was no statistically significant interaction effect between DII and albuminuria across a range of subgroups stratified by gender, age, BMI, diabetes, hypertension, and eGFR (*P* for interaction > 0.05) (Figure 3).

## Discussion

Our study found an association between the higher DII and the increased probability of albuminuria after adjusting for various potential confounders in participants through a cross-sectional study of 70,190 participants from NHANES. The odds of albuminuria had a 5% increase with each unit increase in DII. Subgroup analysis and interaction tests confirmed that the findings are consistent and reliable across the different populations. This is the first study to examine the association between DII and albuminuria and identified the positive correlation between DII and albuminuria. Reducing the degree of dietary inflammation may become an intervention to prevent and alleviate abnormal excretion of albuminuria.

The stable positive correlation between DII and albuminuria was confirmed in a fully adjusted model. In a multifactorial logistic regression model, the effects of BMI and waist circumference were notable. This finding is consistent with the study by Qin et al. (26) on weight-adjusted waist circumference index and increased urinary albumin excretion. Hypertension and diabetes also had significant effect in the fully adjusted model. As Guo et al. (27) found that the relationship between DII and chronic kidney disease in participants with type II diabetes, the ACR was 1.41 times greater in the highest DII group than the lowest (T3 vs. T1). Several randomized controlled trials have also demonstrated a worse prognosis for kidney disease patients with diabetes or hypertension (28, 29). Therefore, kidney disease patients with diabetes or hypertension should be given higher priority. In subgroup analysis, none of the interactions were significant across gender, age, BMI, diabetes, hypertension, and eGFR subgroups, indicating that the association between DII and albuminuria in the general population.

Previous studies have reported an association between DII and a variety of renal diseases including CKD and kidney stones by examining different epidemiologic methods and participants (10, 12, 27, 30). A study based on 21,649 adult Americans reported the odds of prevalent CKD increased from 5.3% in the lowest to 9.3% in the highest DII quartile (31). Research enrolled 1,422 Western Australian women confirmed a higher risk of Kidney Related Diseases in the highest quintile of DII group (32). The all-cause mortality increased from 24.04% in the lowest to 31.23% in the highest DII tertile shown in a report based on 4,554 CKD patients (33). A study from Iowa women renal cancer patients revealed a positive association between higher DII scores and renal cancer risk, although there were no significant interactions (34). However, due to the limited inclusion of data, missing data, and substantial heterogeneity in all the above studies, more large-scale, multicenter studies are still needed in the future.

DII is a calculated value that evaluates the degree of inflammation in a participant's diet. The 28 dietary components and nutrients used to calculate DII have been shown in numerous experiments to correlate with the progression of CKD. A randomized controlled trial found that thiamine helped reduce the degree of albuminuria (35). Increased vitamin D intake has been found to slow down the progression of CKD by modulating inflammatory response (36). CKD patients on hemodialysis who were supplemented with omega-3 fatty acids had significantly lower levels of CRP, IL-6, TNF-alpha, and IL-10/IL-6 ratio, reducing cardiovascular risk (37). However, interestingly a study found that elevated serum MUFAs levels in patients during CKD progression may lead to an increased risk of cardiovascular disease, but dietary MUFAs intake had no effect on serum MUFAs levels (38). Beta-carotene inhibits inflammatory genes for protection against Ang II-induced kidney injury (39). Selenium alleviates acute kidney injury induced by ischemia-reperfusion injury (40). Low intake of fiber and potassium and high intake of sugar and cholesterol contribute to the progression of CKD (41). Based on these experimental results, we believe that it is feasible to investigate the association between DII and albuminuria. The use of DII to assess the dietary inflammation among individuals at-risk for albuminuria may identify potential modifiable dietary factors, whereby decreased DII may decrease the odds of albuminuria and CKD prevalence.

Poorly controlled diabetes can lead to decreased eGFR, hyperalbuminuria, and eventual progression to diabetic nephropathy (42). Patients with essential hypertension are more likely to develop albuminuria than the average subject (43). In our subgroup analysis, the OR values were higher in the subgroups with diabetes and hypertension (Diabetes: OR = 1.01; non-Diabetes: OR = 1.07; Hypertension: OR = 1.02; non-Hypertension OR = 1.10). This may be related to the fact that diabetes patients need to take glucose-lowering drugs, and hypertensive patients need to take antihypertensive drugs for a long period. Many commonly used hypoglycemic and antihypertensive drugs have been shown to act as anti-inflammatory agents through different mechanisms. Metformin may act as an anti-inflammatory agent by inhibiting NLRP3 inflammasome activation and IL-1β production in macrophages, as well as inflammasome-independent IL-6 secretion (44). Empagliflozin attenuates chronic inflammation associated with obesity by reducing plasma TNF-α levels (45). Perindopril prevents sepsis-induced acute kidney injury by reducing plasma TNF-α levels (46). ACEIs and ARBs reduce levels of inflammatory markers including IL-6, TNF-α, and CRP (47).

Strengths of our study included the use of a large representation of a number of specimens and the fact that we adjusted for multiple confounders. For example, eGFR is an important indicator for evaluating renal function and excludes the effects of age, gender, and so on. Therefore, we used eGFR as a covariate in our fully adjusted model, and subgrouped participants according to eGFR to make our study more accurate. Although only 28 dietary components and nutrients were included in our study's calculation of DII, previous studies have confirmed that using 28 of the dietary components and nutrients in the calculation of DII is as effective in predicting dietary validation potential as the larger 45 dietary components and nutrients (48). However, there were also some unavoidable limitations of our study, so the revelations of this study should be interpreted with caution. Firstly, the present study being a cross-sectional study, it was not possible to derive a causal relationship, and secondly, we were unable to include all the covariates affecting the outcome and could not completely exclude the influence of other factors; In addition, the presence of memory bias in the questionnaire data in the NHANES database can lead to some random error; and participants with missing DII data in the database were also prone to missing data on both BMI and waist circumference (33), the two data we included as covariates, and thus it may have caused potentially biased. Recently, related studies have found that the energy-adjusted dietary inflammation index (E-DII) mitigates bias due to differences in energy intake (49). In the future, further inclusion of E-DII and large prospective studies or randomized clinical trials are needed to confirm this result.

## Conclusion

Our study demonstrated that elevated DII causing by inappropriate consumption is associated with high odds of albuminuria. While the potential causal relationship between DII and albuminuria needs more investigation. Therefore, controlling DII through dietary modifications may be a novel focus on attenuating the degree of albuminuria.

## Data availability statement

The original contributions presented in the study are included in the article/supplementary material, further inquiries can be directed to the corresponding author.

## Ethics statement

Ethical review and approval was not required for the study on human participants in accordance with the local legislation and institutional requirements. Written informed consent from the participants was not required to participate in this study in accordance with the national legislation and the institutional requirements.

## Author contributions

LD: Validation, Visualization, Writing – original draft, Writing – review & editing, Project administration. HG: Conceptualization, Resources, Supervision, Writing – review & editing. CZ: Investigation, Methodology, Writing – review & editing. BJ: Data curation, Formal analysis, Writing – review & editing. SZ: Data curation, Investigation, Writing – review & editing. JZ: Formal analysis, Software, Writing – review & editing.
